# Sensitive western blotting for detection of endogenous Ser129-phosphorylated α-synuclein in intracellular and extracellular spaces

**DOI:** 10.1038/srep14211

**Published:** 2015-09-18

**Authors:** Asuka Sasaki, Shigeki Arawaka, Hiroyasu Sato, Takeo Kato

**Affiliations:** 1 Department of Neurology, Hematology, Metabolism, Endocrinology and Diabetology, Yamagata University Faculty of Medicine. 2-2-2 Iida-nishi, Yamagata 990-9585, Japan

## Abstract

α-Synuclein deposited in Lewy bodies, a pathological hallmark of Parkinson’s disease (PD), is highly phosphorylated at serine 129 (Ser129). In contrast, there is very little Ser129-phosphorylated α-synuclein in the normal brains. This difference suggests that Ser129-phosphorylation is involved in neurodegenerative processes of PD. However, the role of this modification remains unclear. One limiting factor for relevant biochemical analyses is that it is difficult to detect endogenous Ser129-phosphoryated α-synuclein by western blotting, because α-synuclein monomers detached from the transferred membrane during incubation. Here, we reported that combination fixation of the transferred membrane with 4% paraformaldehyde and 0.01 ~ 0.1% glutaraldehyde produced an approximately 10-fold increase in the sensitivity for Ser129-phosphorylated α-synuclein monomers, allowing detection of endogenous proteins even in conditioned medium, human cerebrospinal fluid, and extracts from cell lines and human brain. This method may enable more detailed biochemical analyses for α-synuclein transmission between intra and extracellular spaces under physiological and pathological conditions.

The pathological hallmarks of Parkinson’s disease (PD) are loss of dopaminergic neurons in the substantia nigra pars compacta, and the appearance of insoluble aggregates of α-synuclein, called Lewy bodies (LBs) and Lewy neurites, in the surviving nigral neurons^1^,^2^,^3^. Approximately 90% of α-synuclein deposited in LBs is phosphorylated at serine 129 (Ser129)^4^,^5^. In contrast, only 4% or less of the total α-synuclein is phosphorylated at this residue in brains from individuals without PD^4^,^5^. This disparity suggests that levels of Ser129-phosphorylated α-synuclein are tightly regulated by kinases, phosphatases, and degradation pathways under physiological conditions, and extensive Ser129-phosphorylation occurs concurrently with LB formation and dopaminergic neurodegeneration. In a *Drosophila* model of PD, co-expression of α-synuclein and *Drosophila* G-protein-coupled receptor kinase 2 (Gprk2), an ortholog of G-protein-coupled receptor kinase (GRK) 4/5/6 6, generated Ser129-phosphorylated α-synuclein and enhanced α-synuclein toxicity^7^. In a rat recombinant adeno-associated virus (rAAV)-based model, co-expression of A53T α-synuclein and human GRK6 accelerated α-synuclein-induced degeneration of dopaminergic neurons^8^. Conversely, co-expression of wild-type α-synuclein and Polo-like kinase 2 (PLK2) attenuated loss of dopaminergic neurons^9^. This study also showed that phosphorylation and binding of PLK2 resulted in autophagic clearance of α-synuclein proteins^9^. Although these findings raise the possibility that Ser129-phosphorylation modulates the metabolic fate and toxicity of α-synuclein, the physiological and pathological roles of Ser129-phosphorylation remain unclear.

Analyses of endogenous Ser129-phosphorylated α-synuclein are necessary in order to avoid erroneous conclusions regarding α-synuclein overexpression. However, it is generally difficult to detect endogenous Ser129-phosphorylated α-synuclein in cell lines and human brain tissues using conventional western blotting^10^,^11^. Lee and Kamitani reported a simple but effective improvement in the western blotting technique and demonstrated that endogenous levels of total α-synuclein monomers, including phosphorylated and non-phosphorylated forms, could be detected in cell lines and mouse tissues by fixing the transferred membrane with 0.4% paraformaldehyde^11^. They also observed that paraformaldehyde fixation blocked detachment of the target protein from the transferred membrane during incubation, resulting in signal enhancement^11^. However, detection of endogenous Ser129-phosphorylated α-synuclein remains difficult^11^. In the present study, we investigated the optimal concentration of paraformaldehyde to detect endogenous total α-synuclein. In addition, previous studies reported that the fixation of the transferred membrane with glutaraldehyde enhanced the signals, such as calmodulin, S100β^12^, metallothioneins-1 (MT-1), MT-2, and MT-3^13^. We tested the effects of glutaraldehyde on detection of endogenous Ser129-phosphorylated α-synuclein. Here, we report that fixation of the transferred membrane with 4% paraformaldehyde more effectively detects endogenous total α-synuclein monomers than fixation with 0.4% paraformaldehyde. A combination of 4% paraformaldehyde and 0.01 ~ 0.1% glutaraldehyde improved detection of endogenous Ser129-phosphorylated α-synuclein monomers in extracts from cell lines and human brains. This modified method also enabled us to visualize the signals from endogenous Ser129-phosphorylated α-synuclein monomers in conditioned medium (CM) and human cerebrospinal fluid (CSF). The observed signal enhancement was seen in both α-synuclein monomers and other proteins, despite variations in molecular size, although the effectiveness and optimal concentration of paraformaldehyde or glutaraldehyde differed for each protein or primary antibody.

## Results

### Concentration dependent effect of membrane fixation with paraformaldehyde on detection of total and Ser129-phosphorylated α-synuclein monomers by western blotting

**Figure 1:**
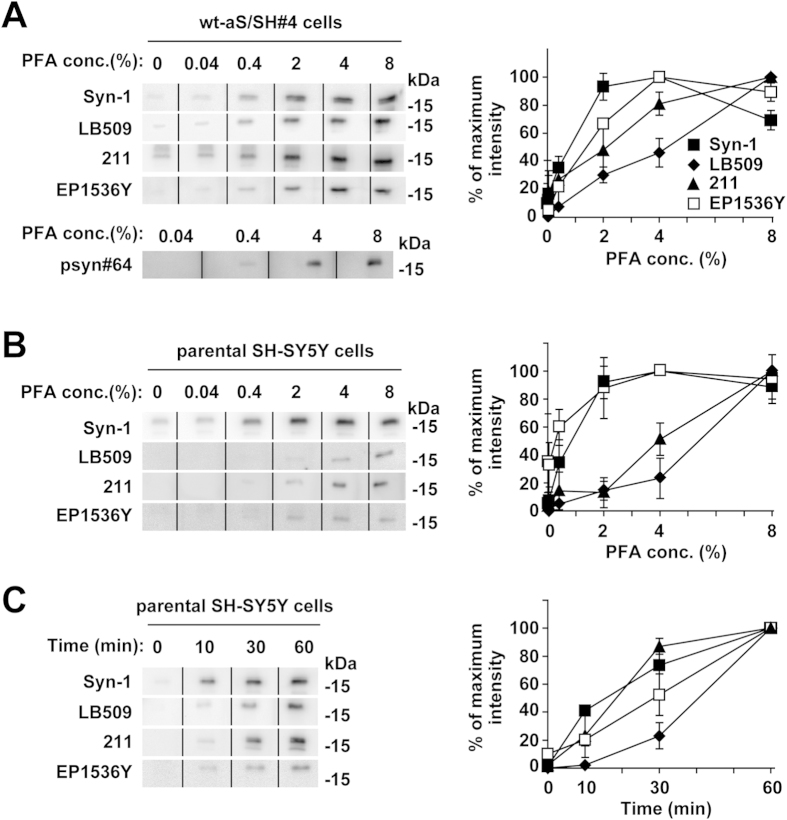
The concentration dependent effect of transferred membrane fixation with paraformaldehyde on α-synuclein monomer detection by western blotting (WB). After SDS-PAGE, the transferred membrane was treated with PBS containing the indicated concentrations of paraformaldehyde for 30 min (**A**,**B**) or 4% paraformaldehyde for indicated durations (**C**). For the membrane without paraformaldehyde fixation, this step was omitted, and was immediately followed by the blocking step. It should be noted that WB with anti-β-actin antibody as a loading control was not performed because the β-actin signals were altered by the fixation. Although equal amounts of protein samples were simultaneously loaded on the gel, the transferred membrane was cut into several strips on the protein marker lanes between sample lanes to test fixation conditions. The strips were spliced and reconstituted to be one original membrane according to the protein markers, and then we simultaneously detected signals by CCD camera. Solid lines indicated cropping boundaries of strips (please note that this editing is done in the blot panels in Figs 2, 3, 4, 5). (**A**) Cell lysates (5 μg/lane) of SH-SY5Y cells stably expressing wild-type α-synuclein (wt-aS/SH#4) were loaded on SDS-PAGE and analyzed by WB with anti-total α-synuclein antibody (Syn-1, 211, or LB509). WB was also performed with anti-Ser129 phosphorylated α-synuclein specific antibody (EP1536Y or psyn#64). (**B**) Cell lysates (10 μg/lane) of parental SH-SY5Y cells were analyzed by WB with the same set of antibodies, except psyn#64. (**C**) Time dependent effect of the 4% paraformaldehyde fixation. Cell lysates (10  g/lane) of parental SH-SY5Y cells were analyzed by WB with Syn-1, 211, LB509, or EP1536Y. The transferred membrane was treated with the fixation solution for 10, 30, or 60 min. Left panels show representative blots. The graphs shows the signal intensity (means ± SD) as a percentage of the maximum observed on the blots.

To assess whether transferred membrane fixation with paraformaldehyde improves sensitivity to detect total α-synuclein monomers, including non-phosphorylated and phosphorylated forms, in a paraformaldehyde concentration dependent manner, we compared the signals for total α-synuclein monomers with increasing paraformaldehyde concentrations. When extracts from SH-SY5Y cells stably expressing wild-type α-synuclein (wt-aS/SH#4) were analyzed by western blotting with anti-α-synuclein monoclonal antibody (Syn-1), the total α-synuclein monomer signals were enhanced by membrane fixation with paraformaldehyde in a concentration dependent manner, up to 4% paraformaldehyde (Fig. 1A, Supplementary figure 1). Fixation with 4% paraformaldehyde produced a 3-fold increase in signal, compared with 0.4% paraformaldehyde fixation. Western blotting using anti-human α-synuclein monoclonal antibody (LB509) also resulted in signal enhancement with high concentrations of paraformaldehyde, although to a lesser extent (Fig. 1A, Supplementary figure 1). Western blotting with anti-human α-synuclein monoclonal antibody (211) demonstrated that the signals for total α-synuclein monomers were enhanced by membrane fixation with paraformaldehyde in a concentration dependent manner, similar to the effects observed for LB509 antibody (Fig. 1A, Supplementary figure S1). Next, we tested whether this method produced similar results for detection of Ser129-phosphorylated α-synuclein. Western blotting using rabbit monoclonal antibody (EP1536Y), which is specific to Ser129-phosphorylated α-synuclein, produced Ser129-phosphorylated α-synuclein monomer signals that were enhanced by increasing paraformaldehyde concentrations up to 4% (Fig. 1A, Supplementary figure S1). The 4% paraformaldehyde fixation condition produced a 4-fold increase in the signals compared to 0.4% paraformaldehyde fixation. The Ser129-phosphorylated α-synuclein signals were also enhanced by increasing the paraformaldehyde concentration when different anti-Ser129-phosphorylated α-synuclein monoclonal antibody, psyn#64 was used (Fig. 1A, Supplementary figure S1).

To elucidate whether fixation with a higher concentration of paraformaldehyde more effectively detects endogenous α-synuclein monomers, parental SH-SY5Y cells lysates were analyzed by western blotting. Western blotting with Syn-1 antibody and 4% paraformaldehyde fixation detected endogenous total α-synuclein monomers more clearly, compared with the conventional western blotting method without fixation (Fig. 1B, Supplementary figure S1). Signals for endogenous total α-synuclein monomers were enhanced by increasing paraformaldehyde concentrations with 211 or LB509 antibody (Fig. 1B, Supplementary figure S1). In addition, western blotting with EP1536Y antibody demonstrated that 4% paraformaldehyde fixation enabled detection of endogenous Ser129-phosphorylated α-synuclein monomers, although these signals were weak (Fig. 1B, Supplementary figure S1).

To test time dependency of the paraformaldehyde fixation on detection of endogenous α-synuclein monomers, we treated the transferred membrane with 4% paraformaldehyde for 10, 30, or 60 min. Western blotting of parental SH-SY5Y cell lysates with Syn-1, 211, or LB509 antibody demonstrated that the signals were increased in a time dependent manner (Fig. 1C, Supplementary figure S1). Western blotting with EP1536Y antibody also showed the time dependent enhancement of signals for endogenous Ser129-phosphorylated α-synuclein monomers (Fig. 1C, Supplementary figure S1).

### Effect of membrane fixation with glutaraldehyde on α-synuclein monomer detection in western blotting

**Figure 2:**
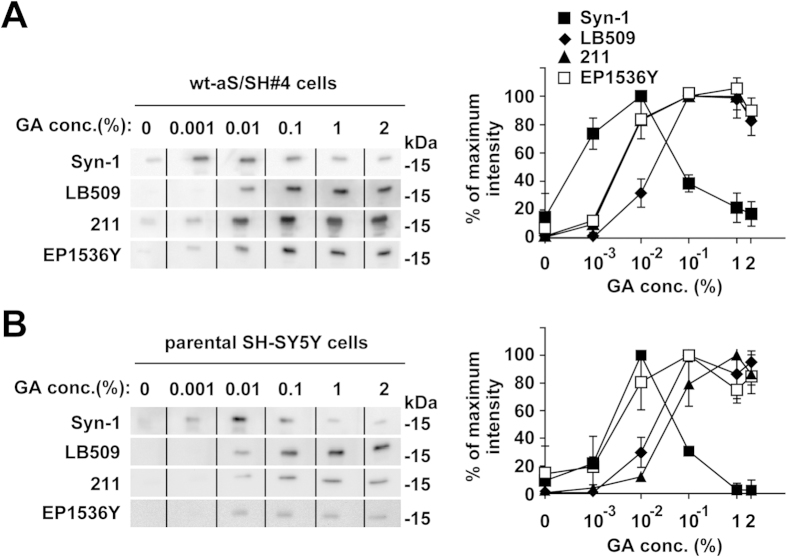
The concentration dependent effect of transferred membrane fixation with glutaraldehyde on α-synuclein monomer detection by WB. The transferred membrane was treated with PBS containing 0.001%, 0.01%, 0.1%, 1%, or 2% glutaraldehyde for 30 min. WB with anti-β-actin antibody as a loading control was not performed, because the β-actin signals were altered by the fixation. (**A**) Cell lysates (5 μg/lane) of wt-aS/SH#4 cells were loaded on SDS-PAGE and analyzed by WB with Syn-1, 211, LB509, or EP1536Y antibody. (**B**) Cell lysates (10 μg/lane) of parental SH-SY5Y cells were analyzed by WB with the same set of antibodies. Left panels show representative blots. The graph shows the signal intensity (means ± SD) as a percentage of the maximum observed on the blots.

Next, we tested whether fixation of the transferred membrane with glutaraldehyde affects α-synuclein detection. Western blotting using wt-aS/SH#4 cell lysates with Syn-1 antibody demonstrated that glutaraldehyde fixation enhanced the signals for α-synuclein monomers at low concentrations of 0.001 ~ 0.01% (Fig. 2A, Supplementary figure S2). Higher concentrations of glutaraldehyde diminished the signals. In contrast, western blotting with 211 or LB509 antibody demonstrated that glutaraldehyde fixation enhanced the signals in a concentration dependent manner, up to 0.1% glutaraldehyde (Fig. 2A, Supplementary figure S2). Western blotting with glutaraldehyde fixation using EP1536Y antibody remarkably enhanced the signals for Ser129-phopshorylated α-synuclein monomers, similar to effects with LB509 antibody (Fig. 2A, Supplementary figure S2). Western blotting of parental SH-SY5Y cells with Syn-1 antibody resulted in endogenous total α-synuclein monomer signals that peaked at 0.01% glutaraldehyde fixation (Fig. 2B, Supplementary figure S2). Experiments with 211 or LB509 antibody demonstrated that glutaraldehyde fixation enhanced the signals for endogenous total α-synuclein monomers in a concentration dependent manner, up to 0.1 ~ 1% (Fig. 2B, Supplementary figure S2). Furthermore, western blotting with EP1536Y antibody demonstrated that glutaraldehyde fixation increased the signals for endogenous Ser129-phosphorylated α-synuclein monomers (Fig. 2B, Supplementary figure S2). The optimal concentrations of glutaraldehyde were 0.01 or 0.1%.

### Effect of combination fixation with paraformaldehyde and glutaraldehyde on α-synuclein monomer detection in western blotting

**Figure 3:**
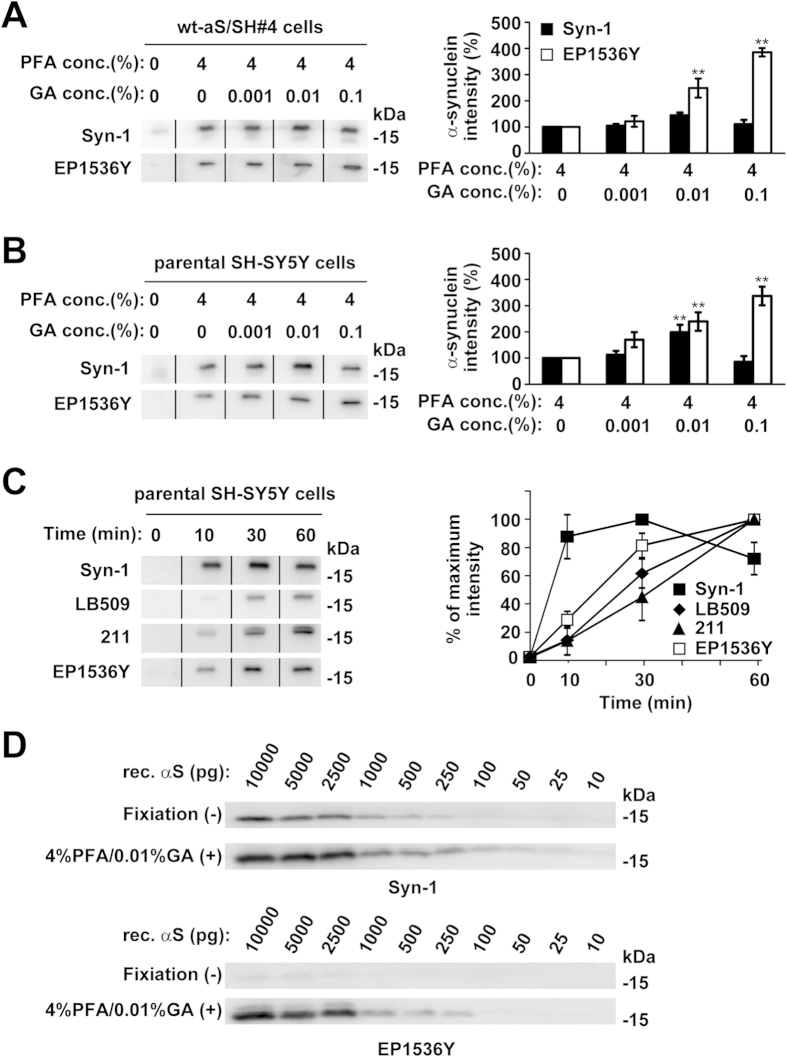
The effect of paraformaldehyde and glutaraldehyde combination on α-synuclein monomer detection by WB. (**A**) Effect of combination fixation for overexpressed α-synuclein detection. Cell lysates (5 μg/lane) of wt-aS/SH#4 cells were loaded on SDS-PAGE and analyzed by WB with Syn-1 or EP1536Y antibody. The transferred membrane was treated with PBS containing 4% paraformaldehyde and 0%, 0.001%, 0.01%, or 0.1% glutaraldehyde for 30 min. For the membrane without paraformaldehyde and glutaraldehyde fixation, this step was omitted, and was immediately followed by the blocking step. *B*. Effect of combination fixation for endogenous α-synuclein detection. Cell lysates (10 μg/lane) of parental SH-SY5Y cells were analyzed by WB with Syn-1 or EP1536Y. The graphs of (**A**,**B**) show the signal intensity (means ± SD) as a percentage of the 4% paraformaldehyde single fixation. *P* values were estimated by one-way ANOVA with a Bonferroni correction (**P < 0.01). In the Syn-1 signals for overexpressed total α-synuclein, *P* values were estimated by one-way ANOVA with the Games-Howell post hoc test, because variances were unequal. (**C**) Time dependent effect of combination fixation with 4% paraformaldehyde and 0.01% glutaraldehyde. Cell lysates (10 μg/lane) of parental SH-SY5Y cells were analyzed by WB with Syn-1, 211, LB509, or EP1536Y antibody. The transferred membrane was treated with combination fixation solution for 10, 30, or 60 min. Left panels show representative blots. The graph of (**C**) shows the signal intensity (means ± SD) as a percentage of the maximum observed on the blots. (**D**) Comparison of the sensitivity of α-synuclein monomer detection between the conventional and combination fixation methods. After partially phosphorylating E. coli derived recombinant α-synuclein at Ser129 by casein kinase 2 (CK2), the indicated amounts of proteins were analyzed by WB with Syn-1 or EP1536Y. The transferred membranes were treated with or without combination fixation with 4% paraformaldehyde and 0.01% glutaraldehyde. Note that phosphorylated proteins contain non-phosphorylated ones, so that they are shown as amounts of total proteins.

To assess whether glutaraldehyde fixation additively enhances the signals for total and Ser129-phoshorylated α-synuclein monomers, we investigated the effects of glutaraldehyde addition on signal detection in wt-aS/SH#4 cells and parental SH-SY5Y cells. Paraformaldehyde fixation was set at a constant concentration of 4%. In western blotting with Syn-1 antibody, this constant 4% paraformaldehyde fixation enabled us to detect signals representing overexpressed and endogenous total α-synuclein monomers (Fig. 3A,B, Supplementary figure S3). When we compared the signals for α-synuclein monomers in combination fixation with paraformaldehyde and glutaraldehyde to those in single fixation with paraformaldehyde, there was no significant change in the signals for overexpressed total α-synuclein monomers by adding 0.01% glutaraldehyde (144.0 ± 11.3%, n = 3, *P *= 0.053) (Fig. 3A, Supplementary figure S3). The signals for endogenous α-synuclein monomers were significantly enhanced by adding 0.01% glutaraldehyde (198.7 ± 27.9%, n = 3, *P *= 0.001) (Fig. 3B, Supplementary figure S3). Western blotting with EP1536Y antibody demonstrated that signals for overexpressed and endogenous Ser129-phosphorylated α-synuclein monomers were enhanced by adding glutaraldehyde in a concentration dependent manner, up to 0.1% (Fig. 3A,B, Supplementary figure S3). The signals for overexpressed Ser129-phosphorylated α-synuclein monomers were significantly enhanced by adding 0.01% glutaraldehyde (248.7 ± 36.3%, n = 3, *P *< 0.001) and 0.1% glutaraldehyde (235.7 ± 15.9%, n = 3, *P *< 0.001) (Fig. 3A, Supplementary figure S3). The signals for endogenous Ser129-phosphorylated α-synuclein monomers were significantly enhanced by adding 0.01% glutaraldehyde (239.7 ± 35.2%, n = 3, *P *= 0.002) and 0.1% glutaraldehyde (337.0 ± 35.3%, n = 3, *P *< 0.001) (Fig. 3B, Supplementary figure S3). These findings indicate that glutaraldehyde plus paraformaldehyde fixation produced additive effects on detection of endogenous total and Ser129-phosphorylated α-synuclein monomers. In the present study, combination fixation with 4% paraformaldehyde and 0.01 or 0.1% glutaraldehyde produced the most sensitive detection of endogenous total α-synuclein monomers using Syn-1 antibody, and endogenous Ser129-phosphorylated α-synuclein monomers using EP1536Y antibody.

To test time dependency of combination fixation on detection of endogenous α-synuclein monomers, we treated the transferred membrane with 4% paraformaldehyde and 0.01% glutaraldehyde for 10, 30, or 60 min. Western blotting of parental SH-SY5Y cell lysates with Syn-1, 211, or LB509 antibody demonstrated that the signals were increased in a time dependent manner (Fig. 3C, Supplementary figure S3). The signals by Syn-1 antibody peaked at 30 min. Western blotting with EP1536Y antibody also showed the time dependent enhancement of signals for endogenous Ser129-phosphorylated α-synuclein monomers during incubation (Fig. 3C, Supplementary figure S3). In the present study, we treated the transferred membrane for 30 min with the combination of 4% paraformaldehyde and 0.01% glutaraldehyde, because this seemed to be optimal and convenient for simultaneously detecting the signals for total and Se129-phosphorylated α-synuclein.

To assess sensitivity to detect α-synuclein monomers by membrane fixation, we investigated the signals resulting from western blotting using purified recombinant α-synuclein proteins that were partially phosphorylated by incubation with casein kinase 2 (CK2). However, it should be noted that we were unable to quantify the signals for Ser129-phosphorylated α-synuclein because we used a mixture of proteins, including non-phosphorylated and Ser129-phosphorylated forms, as standards. Western blotting with Syn-1 antibody using the conventional method without fixation detected signal indicating 250 pg of total α-synuclein, whereas combination fixation with 4% paraformaldehyde and 0.01% glutaraldehyde detected signal indicating 25 pg of total α-synuclein (Fig. 3D, Supplementary figure S3). Using EP1536Y antibody, the conventional method faintly visualized Ser129-phosphorylated α-synuclein signals indicating 2,500 or 5,000 pg of total α-synuclein. The combination of 4% paraformaldehyde and 0.01% glutaraldehyde enabled us to detect signal for Ser129-phosphorylated α-synuclein as a component of 250 pg of total α-synuclein (Fig. 3D, Supplementary figure S3). These findings indicate that combination fixation led to an approximately 10-fold increase in the sensitivity to detect both total and Ser129-phosphorylated α-synuclein.

### Effect of paraformaldehyde or glutaraldehyde fixation on detection of other proteins by western blotting

**Figure 4:**
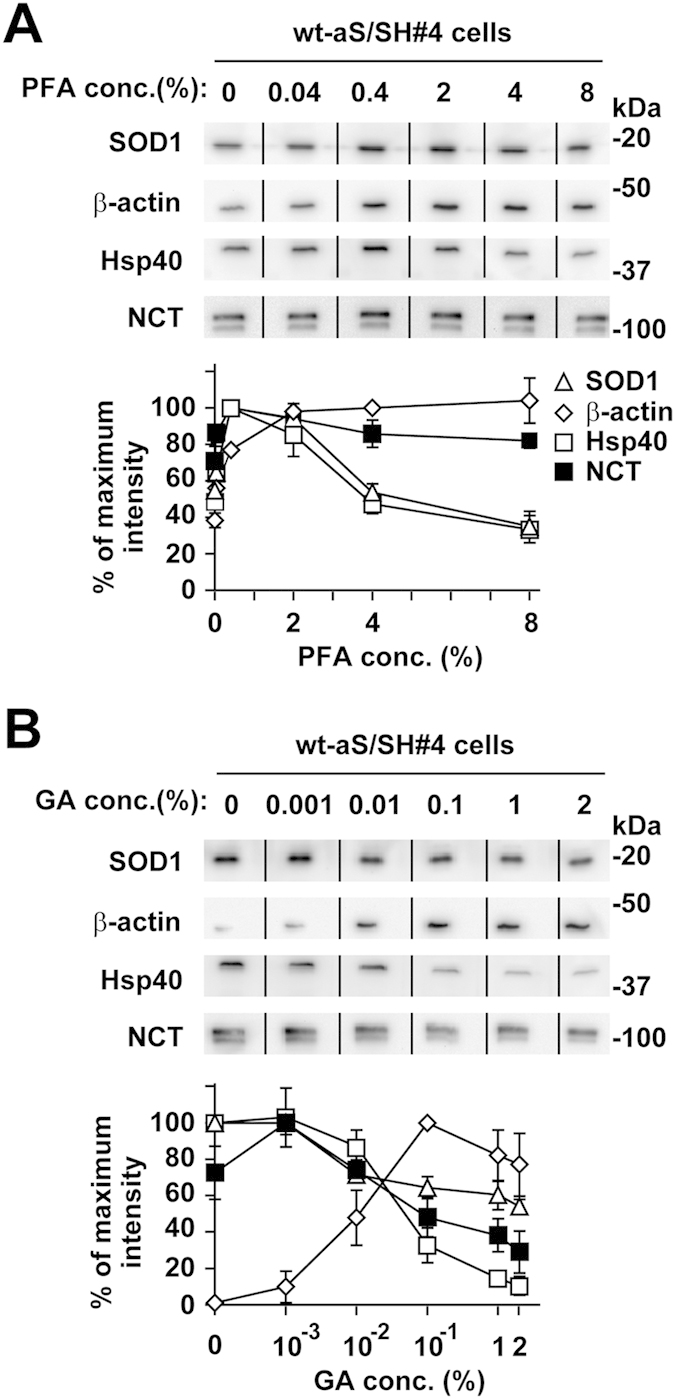
The effect of membrane fixation on detection of other proteins by WB. The cell lysates (5 μg/lane) of wt-aS/SH#4 cells were loaded on SDS-PAGE and analyzed by WB with antibody against either Cu/Zn superoxide dismutase (SOD1), β-actin, heat shock protein 40 (Hsp40), or Nicastrin (NCT). (**A**) Paraformaldehyde fixation. The transferred membrane was treated with PBS containing 0.04%, 0.4%, 2%, 4%, or 8% paraformaldehyde for 30 min. (**B**) Glutaraldehyde fixation. The transferred membrane was treated with PBS containing 0.001%, 0.01%, 0.1%, 1%, or 2% glutaraldehyde for 30 min. For the membrane without paraformaldehyde or glutaraldehyde fixation, this step was omitted, and was immediately followed by the blocking step. Panels show representative blots. The graph shows the signal intensity (means ± SD) as a percentage of the maximum observed on the blots.

To test whether paraformaldehyde fixation effectively detects proteins other than α-synuclein, and whether signal enhancement by paraformaldehyde fixation is influenced by the molecular size of the target molecule, we investigated signals for Cu/Zn superoxide dismutase (SOD1), β-actin, heat shock protein 40 (Hsp40), and nicastrin (NCT) using western blotting with wt-as/SH#4 cell lysates. Signals for all of these proteins were enhanced by paraformaldehyde fixation (Fig. 4A, Supplementary figure S4). However, the effects can be classified into two groups. In one group, signal enhancement peaked at 0.4% paraformaldehyde and signals declined with higher concentrations of paraformaldehyde. This group included SOD1, Hsp40, and NCT (Fig. 4A, Supplementary figure S4). In the second group, signals increased in a paraformaldehyde concentration dependent manner, such as for β-actin (Fig. 4A, Supplementary figure S4).

We then tested the effects of glutaraldehyde fixation on detection of these proteins. The effects were classified into three groups. The first group included proteins for which the glutaraldehyde fixation had no enhancing effect. This result was observed for SOD1 and Hsp40 (Fig. 4B, Supplementary figure S4). The second group demonstrated slightly enhanced signal at a low concentration of 0.001%, and included NCT (Fig. 4B, Supplementary figure S4). The third group had signals that increased in a glutaraldehyde concentration dependent manner up to 0.1% glutaraldehyde, and included β-actin (Fig. 4B, Supplementary figure S4). These findings demonstrate that paraformaldehyde fixation has the potential to enhance signal detection for target proteins of varying molecular sizes. Glutaraldehyde fixation was effective for limited proteins or antibodies, although it sometimes yields remarkable enhancement.

### Specificity of α-synuclein signals detected by paraformaldehyde fixation

**Figure 5:**
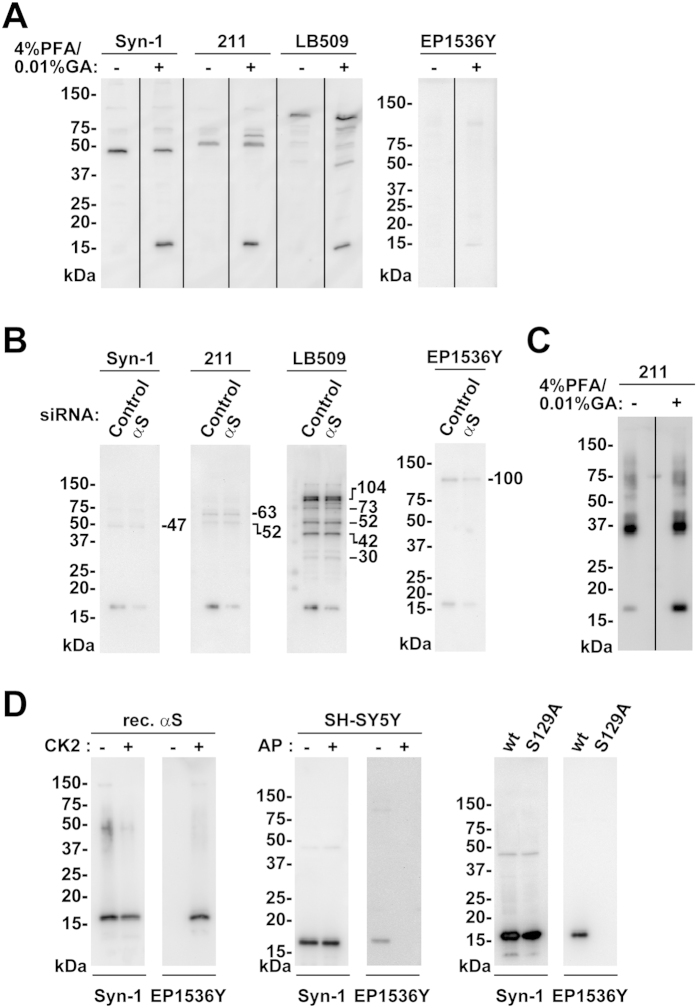
Assessment of α-synuclein signal specificity in paraformaldehyde and glutaraldehyde fixation. The transferred membrane was treated with PBS containing 4% paraformaldehyde and 0.01% glutaraldehyde for 30 min. (**A**) Effects of combination fixation on appearance of α-synuclein monomers and high molecular weight signals by WB with different antibodies. Cell lysates (10 μg/lane) of parental SH-SY5Y cells were loaded on SDS-PAGE and analyzed by WB with either Syn-1, 211, LB509, or EP1536Y antibody. (**B**) Cell lysates (5 μg/lane) of siRNA mediated α-synuclein knockdown or control siRNA SH-SY5Y cells were analyzed by WB with the same set of antibodies. (**C**) Recombinant oligomerized α-synuclein proteins were analyzed by WB with 211 antibody. (**D**) Specificity of Ser129-phosphorylated α-synuclein signals by EP1536Y antibody in combination fixation. In left panels, recombinant α-synuclein (2.5 ng/lane) proteins treated with or without CK2 were analyzed by WB with Syn-1 or EP1536Y antibody. In middle panels, parental SH-SY5Y cell extracts (10 μg/lane) treated with or without calf intestine alkaline phosphatase were analyzed by WB. In right panels, cell lysates (5 μg/lane) of wt-aS/SH#4 or SH-SY5Y cells stably expressing S129A α-synuclein were analyzed by WB.

We assessed the specificity of α-synuclein signals that were enhanced by the paraformaldehyde and glutaraldehyde fixation method. In western blotting of parental SH-SY5Y cells with Syn-1, 211, or LB509 antibody, the combination of 4% paraformaldehyde and 0.01% glutaraldehyde fixation allowed us to detect signals at high molecular weights, in addition to signals corresponding to α-synuclein monomers in each antibody (Fig. 5A, Supplementary figure S5). These included the 63-kDa signal in 211 antibody and multiple signals, such as 73-kDa, 52-kDa, 42-kDa, and 30-kDa ones, in LB509 antibody (Fig. 5A, Supplementary figure S5). In addition, western blotting with EP1536Y antibody demonstrated that the combination of 4% paraformaldehyde and 0.01% glutaraldehyde fixation enhanced the signals migrating at 100-kDa (Fig. 5A, Supplementary figure S5). When we compared the signals of siRNA-mediated α-synuclein knockdown cells with those of control siRNA cells, the Syn-1, 211, and LB509-poitive signals corresponding to α-synuclein monomers were clearly diminished in siRNA knockdown cells to 20.6 ± 5.8% (n = 3, *P *= 0.002), 7.4 ± 3.3% (n = 3, *P *< 0.001), and 9.6 ± 2.0% (n = 3, *P *< 0.001), respectively (Fig. 5B, Supplementary figure S5). However, fixation-enhanced high molecular weight signals showed no significant decreases in siRNA-mediated α-synuclein knockdown cells (Fig. 5B, Supplementary figure S5). The 63-kDa signal in 211 antibody was 92.6 ± 8.1% (n = 3, *P *= 0.251). The 73-kDa, 52-kDa, 42-kDa, and 30-kDa signals in LB509 antibody were 100.1 ± 3.8% (*P *= 0.954), 109.5 ± 8.0% (*P *= 0.176), 90.1 ± 11.4% (*P *= 0.271), and 115.2 ± 12.2% (*P *= 0.097), respectively (n = 3). This finding indicates that combination fixation resulted in non-specific signals at high molecular weights. Western blotting with EP1536Y antibody indicated that signals corresponding to Ser129-phosphorylated α-synuclein monomers were reduced in α-synuclein knockdown cells to 30.3 ± 9.8% (n = 3, *P *= 0.007) (Fig. 5B, Supplementary figure S5). The fixation-enhanced 100-kDa signals were also reduced in siRNA-mediated α-synuclein knockdown cells (38.2 ± 13.1%, n = 3, *P *= 0.015) (Fig. 5B, Supplementary figure S5). We could see the fixation-independent signals, such as the 47-kDa one in Syn-1 antibody, the 52-kDa one in 211 antibody, and the 104-kDa one in LB509 antibody (Fig. 5A, Supplementary figure S5). These signals showed no significant decreases in siRNA knockdown cells (Fig. 5B, Supplementary figure S5). The 47-kDa signals in Syn-1 antibody, the 52-kDa signals in 211 antibody, and the 104-kDa signals in LB509 antibody were 100.7 ± 11.2% (n = 3, *P *= 0.923), 110.8 ± 15.0% (n = 3, *P *= 0.338), and 91.0 ± 6.3% (n = 3, *P *= 0.133), respectively.

To further assess how this combination fixation affects detection of α-synuclein oligomers, we investigated a change in the signal by using *in vitro* oligomerized α-synuclein proteins in western blotting with 211 antibody. The α-synuclein monomer signals were clearly enhanced by the combination of 4% paraformaldehyde and 0.01% glutaraldehyde fixation (Fig. 5C, Supplementary figure S5). The high molecular weight signals were also slightly enhanced by this fixation (Fig. 5C, Supplementary figure S5). This combination fixation was more effective for detecting monomers than oligomers.

We then assessed the specificity of Ser129-phosphorylated α-synuclein signals by EP1536Y antibody in combination fixation. EP1536Y antibody only recognized CK2-incubated recombinant α-synuclein protein (Fig. 5D, Supplementary figure S5). In SH-SY5Y cells, the endogenous α-synuclein monomer signal that EP1536Y antibody recognized was disappeared by alkaline phosphatase treatment (Fig. 5D, Supplementary figure S5). In addition, the monomer signal was not detected in Ser129-phosphorylation-incompetent mutant S129A α-synuclein expressed cells (Fig. 5D, Supplementary figure S5). These findings showed that EP1536Y antibody specifically recognized Ser129-phosphorylated α-synuclein (Fig. 5D, Supplementary figure S5). However, the fixation-enhanced 100-kDa signal in EP1536Y antibody was not recognized by other phosphorylation-independent α-synuclein antibodies, suggesting that this signal was derived from an unknown protein with the similar phosphorylated epitope (Fig. 5A,B, Supplementary figure S5).

### Effect of membrane fixation on detection of endogenous α-synuclein in the extracts of cell lines, rat brains, and human brains

**Figure 6:**
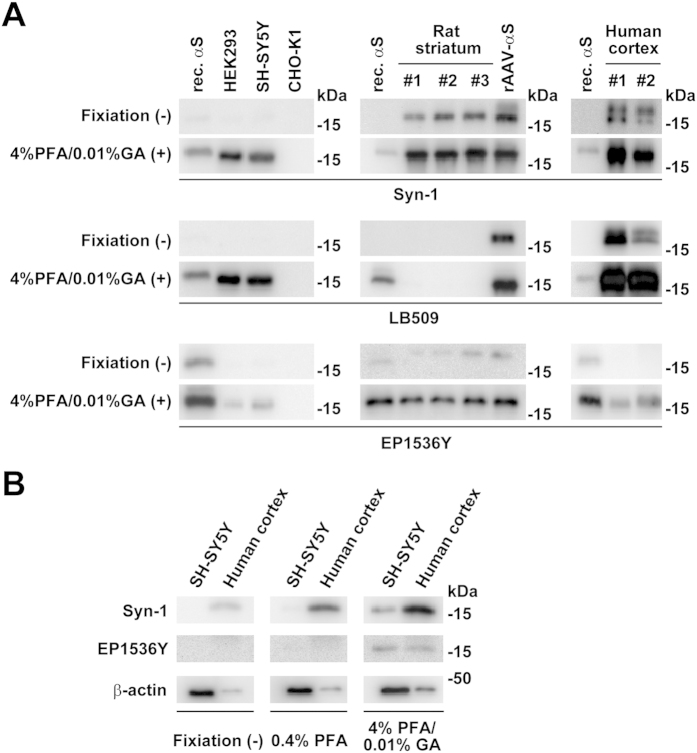
The effect of membrane fixation on detection of endogenous α-synuclein in extracts from cell lines, rat brains, and human brains. The transferred membrane was treated with PBS containing 4% paraformaldehyde and 0.01% glutaraldehyde for 30 min. (**A**) Cell lysates (10 μg/lane) of parental lines of HEK293, SH-SY5Y, and CHO-K1 cells were loaded on SDS-PAGE and analyzed by WB with either Syn-1, LB509, or EP1536Y antibody. Rat striatum extracts (5 μg/lane) were analyzed by WB with the same set of antibodies. As positive controls, recombinant α-synuclein proteins and/or extracts of rat striatum from rats that were injected with rAAV-human α-synuclein were assayed by WB along with samples. Human cerebral cortex extracts (5 μg/lane) were analyzed by WB with the same set of antibodies. (**B**) Comparison of the sensitivity of α-synuclein monomer detection between single 0.4% paraformaldehyde fixation and combination fixation with 4% paraformaldehyde and 0.01% glutaraldehyde. Cell lysates (10 μg/lane) of parental SH-SY5Y cells and extracts (5 μg/lane) of human cerebral cortex were analyzed by WB with Syn-1 or EP1536Y. Panels show representative blots. The same amounts of samples were blotted with anti-β-actin antibody, as a loading control.

To assess how our membrane fixation method applies to biochemical experiments on α-synuclein, we investigated the signals for endogenous α-synuclein in cell lysates of HEK293, SH-SY5Y, and CHO-K1 cells. The conventional method of western blotting using Syn-1 antibody without fixation did not detect signals for endogenous total α-synuclein monomers, whereas the combination of 4% paraformaldehyde and 0.01% glutaraldehyde fixation enabled us to visualize these signals in HEK293 and SH-SY5Y cells (Fig. 6A, Supplementary figure S6). No obvious signals were observed in CHO-K1 cells. Western blotting with EP1536Y antibody indicated that the conventional method did not detect signals for endogenous Ser129-phosphorylated α-synuclein monomers, whereas the combination of 4% paraformaldehyde and 0.01% glutaraldehyde fixation allowed us to detect these signals in HEK293 and SH-SY5Y cells (Fig. 6A, Supplementary figure S6). We then assessed signals for endogenous α-synuclein in rat striatum homogenates. Western blotting with Syn-1 antibody using the conventional method detected signals for endogenous total α-synuclein monomers, whereas the combination of 4% paraformaldehyde and 0.01% glutaraldehyde fixation remarkably enhanced these signals (Fig. 6A, Supplementary figure S6). Western blotting with EP1536Y antibody using the conventional method produced a faint signal for endogenous Ser129-phosphorylated α-synuclein monomers (Fig. 6A, Supplementary figure S6). The combination of 4% paraformaldehyde and 0.01% glutaraldehyde fixation resulted in clearer visualization of these signals (Fig. 6A, Supplementary figure S6). When we examined signals for endogenous total α-synuclein in human cerebral cortex homogenates, western blotting with Syn-1 or LB509 antibody demonstrated that the combination of 4% paraformaldehyde and 0.01% glutaraldehyde fixation further enhanced the signals for endogenous total α-synuclein monomers, compared with the conventional method (Fig. 6A, Supplementary figure S6). Western blotting with EP1536Y antibody using the conventional method did not detect any signals for endogenous Ser129-phosphorylated α-synuclein monomers (Fig. 6A, Supplementary figure S6). However, the combination of 4% paraformaldehyde and 0.01% glutaraldehyde fixation clearly visualized the signals for endogenous Ser129-phosphorylated α-synuclein monomers in the human brain tissue (Fig. 6A, Supplementary figure S6).

To confirm the effects of 4% paraformaldehyde and 0.01% glutaraldehyde combination on endogenous α-synuclein monomer detection, we compared signals yielded by this combination fixation with 0.4% paraformaldehyde fixation, which is the original method described by Lee and Kamitani^11^. Western blotting with Syn-1 antibody using combination fixation enhanced signals for endogenous total α-synuclein monomers in parental SH-SY5Y cells and human cerebral cortex more intensively than 0.4% paraformaldehyde fixation (Fig. 6B, Supplementary figure S6). Western blotting with EP1536Y antibody using 0.4% paraformaldehyde fixation alone did not result in signal detection for endogenous Ser129-phosphorylated α-synuclein monomers in parental SH-SY5Y cells or human cerebral cortex (Fig. 6B, Supplementary figure S6). However, combination fixation enabled detection of signals for endogenous Ser129-phosphorylated α-synuclein monomers (Fig. 6B, Supplementary figure S6). These findings indicate that combination fixation effectively assesses the endogenous expression of Ser129-phosphorylated α-synuclein monomers in extracts from cell lines and mammalian brains.

### Effect of membrane fixation on detection of endogenous α-synuclein in conditioned medium (CM) and cerebrospinal fluid (CSF)

**Figure 7:**
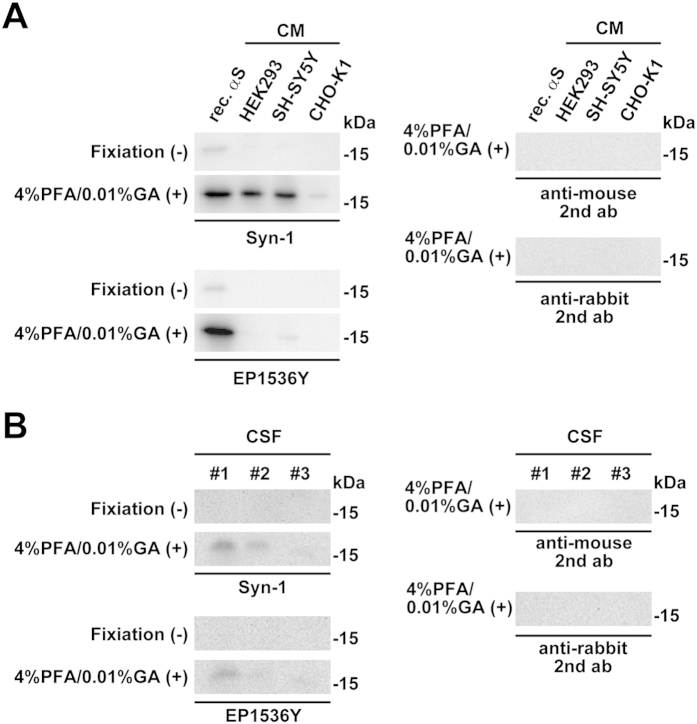
The effect of membrane fixation on detection of endogenous α-synuclein in conditioned medium (CM) and human cerebrospinal fluid (CSF). The transferred membrane was treated with PBS containing 4% paraformaldehyde and 0.01% glutaraldehyde for 30 min. The CM was collected from cultured parental HEK293, CHO-K1, and SH-SY5Y cells. The CM and CSF were condensed by TCA precipitation, and resolved with sample buffer containing 2.5% β-mercaptoethanol. (**A**) 10 μL of CM (1/10 volume of total) was loaded on SDS-PAGE and analyzed by WB with Syn-1 or EP1536Y antibody. To assess signal specificity, the post-fixative transferred membranes were incubated only with anti-mouse or anti-rabbit secondary antibody. As positive controls, recombinant α-synuclein proteins were subjected to WB along with samples. (**B**) 10 μL of human CSF (1/2 volume of total) were analyzed by WB with Syn-1 or EP1536Y antibody. The post-fixative transferred membranes were also incubated only with anti-mouse or anti-rabbit secondary antibody.

To clarify whether the membrane fixation method effectively detects endogenous α-synuclein in the extracellular space, we investigated the signals by western blotting using the CM from parental HEK 293, SH-SY5Y, and CHO-K1 cells. Western blotting with Syn-1 antibody using the conventional method failed to detect the signals for endogenous total α-synuclein monomers, whereas combination fixation with 4% paraformaldehyde and 0.01% glutaraldehyde enabled us to detect the clear signals in the CM obtained from HEK293 and SH-SY5Y cells (Fig. 7A, Supplementary figure S7). By this combination fixation, the endogenous total α-synuclein monomer was also observed as a faint signal in CM from CHO-K1 cells (Fig. 7A, Supplementary figure S7), although the endogenous total α-synuclein monomer was undetectable in cell lysates of CHO-K1 cells (Fig. 6A, Supplementary figure S6). In addition, western blotting with EP1536Y antibody demonstrated that combination fixation enabled us to detect the signals for endogenous Ser129-phosphorylated α-synuclein monomers in CM from SH-SY5Y cells (Fig. 7A, Supplementary figure S7). However, no obvious signals were observed for endogenous Ser129-phosphorylated α-synuclein monomers in the CM from HEK293 and CHO-K1 cells (Fig. 7A, Supplementary figure S7). When the post-fixative transferred membranes were incubated only with secondary antibodies, we found no obvious signals (Fig. 7A, Supplementary figure S7). These findings indicate that the signals detectable in western blotting with the Syn-1 or EP1536Y antibody were not non-specific effects resulting from treatment with the secondary antibodies.

Finally, we tested whether combination fixation with 4% paraformaldehyde and 0.01% glutaraldehyde was applied to detect the signals for endogenous α-synuclein monomers in human CSF. Western blotting with Syn-1 antibody using the conventional method did not detect the signals for endogenous total α-synuclein monomers, whereas the combination fixation enabled us to detect clear signals in CSF (Fig. 7B, Supplementary figure S7). Western blotting with EP1536Y antibody also demonstrated that combination fixation allowed us to detect the signals from endogenous Ser129-phosphorylated α-synuclein monomers in CSF (Fig. 7B, Supplementary figure S7). When the post-fixative transferred membranes were incubated only with our secondary antibodies, there were no obvious signals; therefore, providing evidence for the specificity of signals enhanced by the fixation (Fig. 7B, Supplementary figure S7).

## Discussion

Our data demonstrate that transferred membrane fixation with paraformaldehyde and glutaraldehyde enabled signal detection for endogenous Ser129-phosphorylated α-synuclein monomers by western blotting. Glutaraldehyde had a remarkable effect on detection, and the most effective combination was 4% paraformaldehyde and 0.01 ~ 0.1% glutaraldehyde in western blotting with EP1536Y antibody. The sensitivity of detection was increased approximately 10-fold in recombinant α-synuclein proteins phosphorylated at Ser129. Although the signals for endogenous Ser129-phosphorylated α-synuclein monomers were very faint (lower than the detection limit by conventional western blotting without fixation), this simple method allowed us to reproducibly visualize these signals in cell line lysates (HEK293 and SH-SY5Y cells) and in extracts from the rat striatum and human cerebral cortex. Lee and Kamitani reported that 0.4% paraformaldehyde fixation in western blotting using EP1536Y antibody effectively enabled visualization of signals for endogenous Ser129-phosphorylated α-synuclein monomers in human cell lines, including Daoy, SK-MEL28, MeWo, and WM266-4 cells; however, it did not enable signal detection in HEK293 and SH-SY5Y cells^11^. The 4% paraformaldehyde and 0.01% glutaraldehyde combination fixation further improved signal sensitivity in lysates from HEK293 and SH-SY5Y cells, compared with 0.4% paraformaldehyde fixation. In addition, combination fixation enabled us to detect endogenous Ser129-phosphorylated α-synuclein in CM from SH-SY5Y cells and in human CSF.

We found that our paraformaldehyde or glutaraldehyde fixation method enhanced signals for different molecules (SOD1, β-actin, Hsp40, and NCT), despite their molecular sizes. However, the enhancing effects were classified into two groups. In the first group, the signal enhancing effect peaked at low concentrations of fixative agents (0.4% paraformaldehyde or 0.001% glutaraldehyde), and declined at higher concentrations. Excessive fixation with paraformaldehyde or glutaraldehyde may inhibit epitope accessibility of antibody on the blot membranes. In the second group, the enhancing effects increased in a concentration dependent manner, up to 8% paraformaldehyde or 0.1% glutaraldehyde. These findings suggest that paraformaldehyde or glutaraldehyde fixation effectively enhances signals for proteins other than α-synuclein, and that it is necessary to optimize the fixation effects in each molecule or antibody.

Our findings do not explain the mechanism of action for improved immunoreactivity to α-synuclein antibody. Lee and Kamitani reported that α-synuclein monomers easily detach from the transferred membrane during incubation, using conventional western blotting protocols^11^. Similarly, Newman *et al.* demonstrated that α-synuclein monomer bands disappeared by washing the transferred PVDF membrane overnight in PBS containing 0.1% (v/v) Tween 20^14^. One hypothesis is that paraformaldehyde fixation may generate intermolecular covalent bonds between α-synuclein monomers themselves or with other proteins on the blot membranes, causing firm adherence to the membrane^14^. However, the enhancing effect of paraformaldehyde fixation was observed by western blotting using purified α-synuclein proteins, indicating that this effect is not due to intermolecular bonds between α-synuclein monomers and other proteins. Alternatively, Newman *et al.* demonstrated that treatment of cells or cell lysates with the reducible amine-reactive crosslinker DSP enhanced α-synuclein monomer signals by western blotting^14^. This enhancement occurred even in samples that underwent reductive cleavage of DSP cross-links by β–mercaptoethanol^14^. The authors proposed that DSP/β–mercaptoethanol treatment neutralized positive charges and increased the hydrophobicity of α-synuclein, resulting in enhanced adhesion to the blot membranes^14^. The effects of paraformaldehyde or glutaraldehyde fixation may occur by a mechanism analogous to DSP/β–mercaptoethanol treatment, because paraformaldehyde and glutaraldehyde react with amino groups and increase hydrophobicity^15^. According to this hypothesis, 0.4% paraformaldehyde membrane fixation may be insufficient to reduce the charges on α-synuclein. Furthermore, the strong crosslinking ability of glutaraldehyde may greatly reduce the hydrophilicity of Ser129-phosphorylated α-synuclein monomers, thereby resulting in higher sensitivity than conventional or 0.4% paraformaldehyde fixation methods.

Ser129-phosphorylation is the dominant α-synuclein modification found in LBs and Lewy neuritis^4^,^5^. This suggests that abnormal elevation of Ser129-phosphorylation accelerates α-synuclein aggregation or that it is a secondary reaction to eliminate misfolded and insoluble α-synuclein. However, the exact role of this modification remains unknown. To address this issue, it is necessary to clarify the stage of α-synuclein aggregation at which abnormal phosphorylation acceleration occurs. Expression levels of Ser129-phosphorylated α-synuclein monomers in radioimmunoprecipitation assay-soluble fractions increase prior to detectable LB pathology in the human cingulate and temporal cortices^16^. In addition, the levels of Ser129-phosphorylated α-synuclein in TBS soluble and moderately insoluble fractions increase in brain samples from patients with PD^17^. These findings suggest that increased Ser129-phosphorylation is an early event in the process of α-synuclein aggregation^16^,^17^. Our previous paper reported that Ser129-phosphorylated α-synuclein was targeted to the proteasome degradation pathway, in addition to dephosphorylation^18^. In addition, Oueslati, *et al.* demonstrated that Ser129-phosphorylation by PLK2 induced autophagic α-synuclein clearance^9^. This phenomenon required both phosphorylation at Ser129 and formation of α-synuclein and PLK2 complexes^9^. Although the role of Ser129-phosphorylation remains controversial, these findings suggest that the state of Ser129-phosphorylation affects the metabolic fate of α-synuclein under physiological conditions^9^,^16^,^17^,^18^. In contrast, alterations in Ser129-phosphorylated α-synuclein proteins have been reported as more sensitive biomarkers for diagnosis of PD than total α-synuclein protein^19^,^20^,^21^. A previous report demonstrated that melanoma cells release microvesicles that attach Ser129-phosphorylated α-synuclein on the membranes^22^. These findings suggest that the regulatory system of intracellular Ser129-phosphorylated α-synuclein protein and the associated release mechanism may be involved in the pathological process of PD. To resolve these questions, it is necessary to detect the levels of endogenous total and Ser129-phosphorylated α-synuclein proteins in intra- and extracellular spaces more stably, using western blotting. Combination fixation with paraformaldehyde and glutaraldehyde seems to be a potent method for performing more quantitative biochemical experiments, and may facilitate studies to elucidate these outstanding questions.

Measurement of α-synuclein levels in the blood plasma and CSF is proposed as a useful biomarker for PD diagnosis^19^,^20^,^23^. Ser129-phosphorylated α-synuclein in CSF weakly correlates with the severity of PD and, when combined with total α-synuclein concentrations, contributed to distinguishing PD from multiple system atrophy and progressive supranuclear palsy^23^. In addition, Ser129-phosphorylated α-synuclein in blood plasma is reported to be a more promising diagnostic biomarker than total α-synuclein^19^,^20^. These results were obtained using sandwich ELISA data. The transferred membrane fixation method may be applied to measurement of Ser129-phosphorylated α-synuclein and total α-synuclein using western blotting. This technique provides assessment of antibody specificity and elucidates the state of α-synuclein monomers and multimers by directly visualizing them on the blot membranes, compared to the ELISA technique.

## Methods

### Human samples

In this study, we performed experiments using human CSF samples. Human CSF samples were collected for diagnostic purpose from three non-PD patients in our department. Written informed consent concerning scientific research use was obtained from all patients. The personal data were anonymized. In addition, we used postmortem human brain tissues. These experiments using human CSF and brain tissues were performed in accordance with “Ethical Guidelines for Human Genome and Genetic Analysis Research” by the Ministry of Health, Labour and Welfare, the Ministry of Education, Culture, Sports, Science and Technology, and the Ministry of Economy, Trade and Industry of Japan (the revised version in 2013). They were approved by the Ethical Committee of Yamagata University Faculty of Medicine.

### Cell culture and siRNA transfection

Human dopaminergic neuroblastoma SH-SY5Y cells (ECACC #94030304) were maintained in Ham’s F-12/Eagle’s minimum essential medium (Sigma) supplemented with 15% fetal bovine serum (FBS, Life Technologies, Inc.), 2 mM l-glutamine (Life Technologies, Inc.) and 1× non-essential amino acids (Sigma). HEK293 cells (JCRB Cell Bank #9068) were maintained in Eagle’s minimum essential medium supplemented with 10% FBS and 2 mM l-glutamine. SH-SY5Y cell lines stably expressing wild-type α-synuclein (wt-aS/SH #4) or phosphorylation-incompetent S129A mutant α-synuclein (S129A-aS/SH #17) were used as described previously^18^. CHO-K1 cells (JCRB Cell Bank #IFO50414) were maintained in Ham’s F-12 supplemented with 10% FBS.

Approximately 30% confluent cells in 6-well plates were transfected with siRNA oligonucleotides (final concentration at 10 nM, stealth RNAi, Life Technologies, Inc.) using RNAiMAX reagent (Life Technologies, Inc.) according to the manufacturer’s protocol^24^. We used 25 nucleotide-long siRNAs for knockdown of α-synuclein (5′-GACCAAAGAGCAAGUGACAAAUGUU-3′). As a non-silencing control for stealth siRNAs, the stealth RNAi negative control medium GC (Life Technologies, Inc.) was used. At 72 h after transfection, the medium was discarded, and the cells were re-transfected with siRNA. At 48 h after the second transfection, the cells were harvested for experiments, as described previously^24^.

### Primary antibodies

The following antibodies were used: anti-α-synuclein (Syn-1, mouse monoclonal, which recognizes total α-synuclein, BD Transduction Laboratories), anti-α-synuclein (211, mouse monoclonal, Sigma), anti-human α-synuclein (LB509, mouse monoclonal, Covance), anti-Ser129-phosphorylated α-synuclein (EP1536Y, rabbit monoclonal, Abcam), anti-Ser129-phosphorylated α-synuclein (psyn#64, mouse monoclonal, Wako), anti-β-actin (AC-15, mouse monoclonal, Sigma), anti-Cu/Zn superoxide dismutase (SOD1; SOD1-100, rabbit polyclonal, Stressgen), anti-nicastrin (NCT; #16420, mouse monoclonal, BD Transduction Laboratories), and anti-heat shock protein 40 (Hsp40; SPA-400, rabbit polyclonal, Stressgen).

### Preparation of protein extracts, conditioned medium, and cerebrospinal fluid

For preparation of cell lysates, cultured cells were suspended in buffer A (20 mM Tris-HCl, pH 7.4, 150 mM NaCl, 1% Triton X-100, 10% glycerol, 1× protease inhibitor cocktail [Roche Diagnostic], 1 mM EDTA, 5 mM NaF, 1 mM Na_3_VO_4_, 1× phosSTOP [Roche Diagnostic]) and kept on ice for 30 min. After centrifugation at 12,000 × g for 30 min, the resulting supernatant was collected and stored at −80 °C until it was used in experiments. To dephosphorylate endogenous phosphorylated α-synuclein, we treated cell lysates with 10 units of calf intestine alkaline phosphatase (Roche Diagnostic) for 2 hour at 37 °C. Cell lysates were obtained by sonication in Tris-buffered saline (TBS, 25 mM Tris-HCl, pH 7.4, 137 mM NaCl, 2.7 mM KCl) and centrifugation at 12,000 × g for 30 min. To quench the reaction, an equal volume of 2 × sample buffer was quickly added into samples followed by heating at 95 °C for 5 min. Rat and human brains were homogenized with a Potter homogenizer (15 strokes) in buffer A. After centrifugation at 1,000 × g for 10 min, the supernatant was further centrifuged at 100,000 × g for 60 min. The resulting supernatant was collected and stored at −80 °C. Protein concentrations were measured by the BCA assay (Thermo Scientific).

For preparation of the conditioned medium (CM), cultured cells were plated onto 10 cm dishes. When cells were 90% confluent, we exchanged the growth media with 6 mL of Opti-MEM (Life Technologies, Inc.). After further 24 h incubation, CM was collected and centrifuged at 1,000 × g for 5 min to remove cell debris. Immediately, 6 mL of CM was added with 1/4 volume of 100% trichloroacetic acid (TCA), incubated for 30 min on ice, and centrifuged at 14,000 × g for 5 min. The resultant pellet was washed three times with 300 μL of cold acetone, air dried, and dissolved in 100 μL of Laemmli’s sample buffer containing 2.5% β-mercaptoethanol. Similarly, after centrifugation at 1,000 × g for 5 min, 1 mL of cerebrospinal fluid (CSF) from non-PD cases was condensed with TCA precipitation and dissolved in 20 μL of sample buffer containing 2.5% β-mercaptoethanol.

### *In vitro* oligomerization of Escherichia coli derived recombinant human α-synuclein proteins

E. coli-derived recombinant human α-synuclein proteins were prepared as described previously^25^. Phosphorylated α-synuclein proteins were obtained by incubating 100 μg of non-phosphorylated protein in buffer (20 mM Tris-HCl, pH 7.5, 50 mM KCl, 10 mM MgCl_2_, 200 μM ATP) containing 1,000 units of recombinant casein kinase 2 (CK2) protein (New England Biolabs)^18^,^24^. The α-synuclein oligomers were prepared by incubating non-phosphorylated proteins in phosphate buffered saline (PBS, 2.97 mM Na_2_HPO_4_·7H_2_O, 1.06 mM KH_2_PO_4_, 155 mM NaCl) containing 20 μM ditiobis (succinimidylpropionate) (DSP) (Thermo Scientific) and 1 × protease inhibitor cocktail at room temperature (RT) for 30 min^26^. The reaction was quenched by addition of 1 M Tris-HCl, pH 7.5 (final concentration 50 mM) and incubated at RT for 15 min. After adding an equal volume of buffer (6 M urea, 2% SDS), samples were sonicated at 30 W for 3 s × 5 times, followed by centrifugation at 100,000 × g for 1 h. The resulting supernatant was collected and stored at −80 °C.

### Western blotting

Samples were denatured at 95 °C for 5 min in Laemmli’s sample buffer containing 2.5% β-mercaptoethanol. Equal protein amounts of denatured samples were subjected to SDS-PAGE on 13.5% polyacrylamide gels. After electrophoresis, gels were incubated in Towbin blotting buffer (25 mM Tris-HCl, 192 mM glycine, 20% methanol) with mild agitation for 30 min. Gels were then transferred to PVDF membranes (0.45 μm pore size, Immobilon-P, Millipore) using the Mini Trans-Blot electrophoretic transfer cell (Bio-Rad) at 250 mA constant current for 1.5 h, using a Model 200/2.0 Power Supply (Bio-Rad). During this step, the apparatus was placed on ice and the buffer was stirred with a magnetic bar. Then, the transferred membrane was incubated in PBS containing the indicated concentration of paraformaldehyde for 30 min, unless the otherwise stated. When we assessed the effects of glutaraldehyde, the indicated concentration of glutaraldehyde was also added to the fixative solution at this stage of the experiment. After incubation, the membrane was washed in TBS containing 0.05% (v/v) Tween 20 (TBS-T) for 10 min 3 times. The membrane was incubated in TBS-T containing 5% skim milk for 30 min. For our conventional western blotting technique, the fixative treatment was omitted. The membrane was further incubated in TBS-T containing 2.5% skim milk and primary antibody overnight in the cold room, and then washed in TBS-T for 10 min 3 times. The membrane was incubated in the same buffer containing the corresponding secondary antibody overnight in the cold room, and washed in TBS-T for 10 min 3 times. When we detected phosphorylated α-synuclein, 50 mM NaF was added to TBS-T containing skim milk^18^. To visualize the signal, membranes were exposed to ECL plus (Thermo Scientific) for detection of total and phosphorylated α-synuclein or Supersignal West Pico chemiluminescent substrate (Thermo Scientific) for other proteins. Signals were recorded using a CCD camera, VersaDog 5000 (Bio-Rad). Levels of total α-synuclein and Ser129-phosphorylated α-synuclein were estimated by measuring band intensities with Quantity One software (Bio-Rad)^18^.

As a positive control for endogenous α-synuclein in rat brain tissue, we used the rat striatum expressing A53T human α-synuclein, using a rAAV vector, as described previously^8^. 1% Triton X-100 soluble extracts were prepared using the method above and loaded to SDS-PAGE.

### Statistical analysis

Comparison between two groups was performed by unpaired Student’s t-test. Multiple comparison was performed by one-way analysis of variance (ANOVA) with a Bonferroni correction. When variances were unequal (the case of Syn-1-positive signals for overexpressed total α-synuclein in combination fixation), the Games-Howell post hoc test was used.

## Additional Information

**How to cite this article**: Sasaki, A. *et al.* Sensitive western blotting for detection of endogenous Ser129-phosphorylated α-synuclein in intracellular and extracellular spaces. *Sci. Rep.* **5**, 14211; doi: 10.1038/srep14211 (2015).

## Supplementary Material

Supplementary Information
