# Supplementary material for: Sensitive western blotting for detection of endogenous Ser129-phosphorylated α-synuclein in intracellular and extracellular spaces
Source: Sci Rep. 2015 Sep 18;5:14211. doi: 10.1038/srep14211 (PMC4585644; doi:10.1038/srep14211)
Supplement: Supplementary Information [file srep14211-s1.pdf]

Supplementary information

Sensitive western blotting for detection of endogenous Ser129-phosphorylated α-synuclein in intracellular and extracellular spaces

Asuka Sasaki[1], Shigeki Arawaka[1], Hiroyasu Sato[1] & Takeo Kato[1]

[1]From the Department of Neurology, Hematology, Metabolism, Endocrinology and Diabetology, Yamagata University Faculty of Medicine. 2-2-2 Iida-nishi, Yamagata 990-9585, Japan

Supplementary figures S1-7

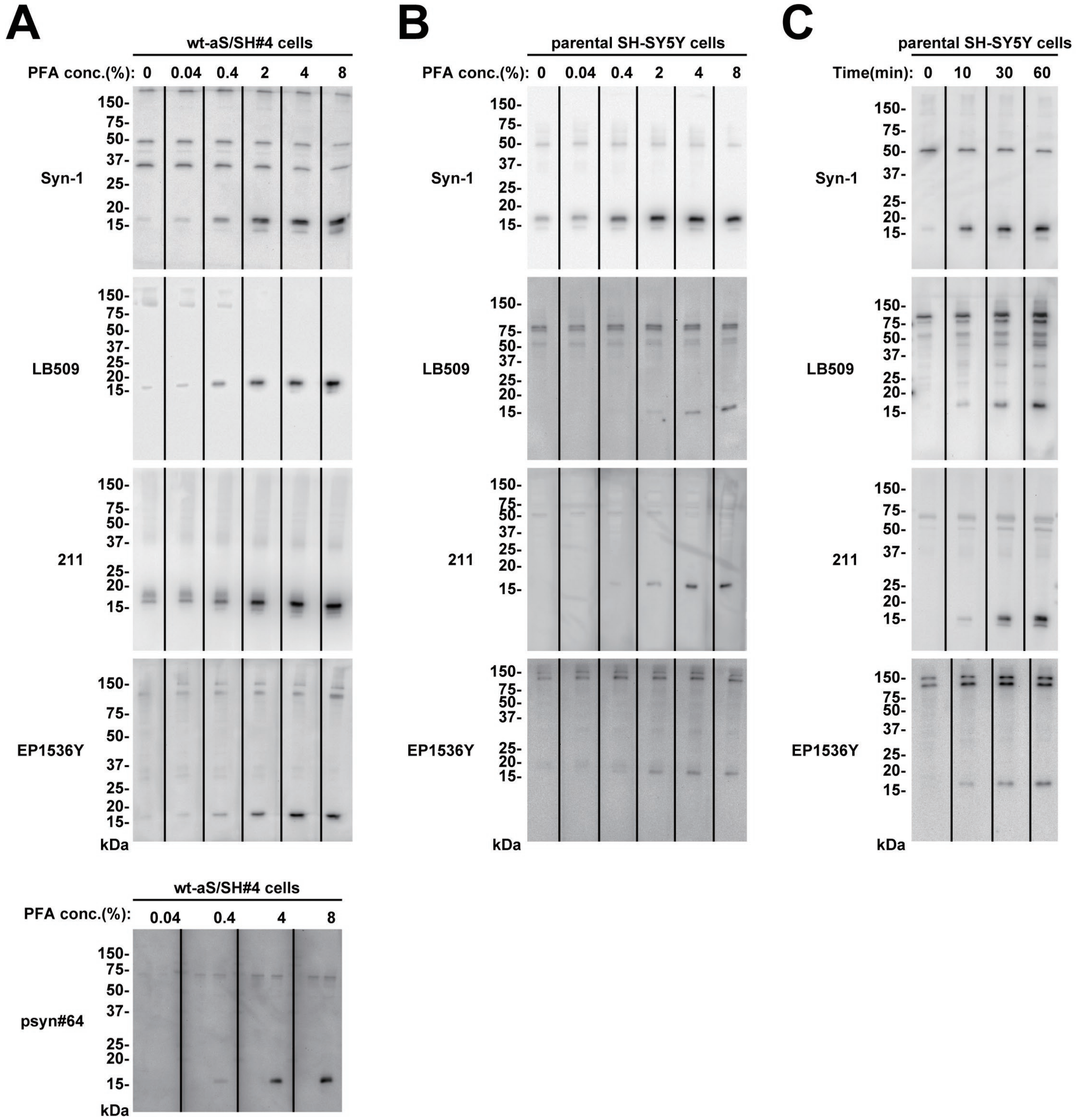

Supplementary figure S1. Full-length images of figure 1. The origin of these panels is identical with figure 1 showing western blotting with Syn-1, LB509, 211, EP1536Y, or psyn#64 antibody.

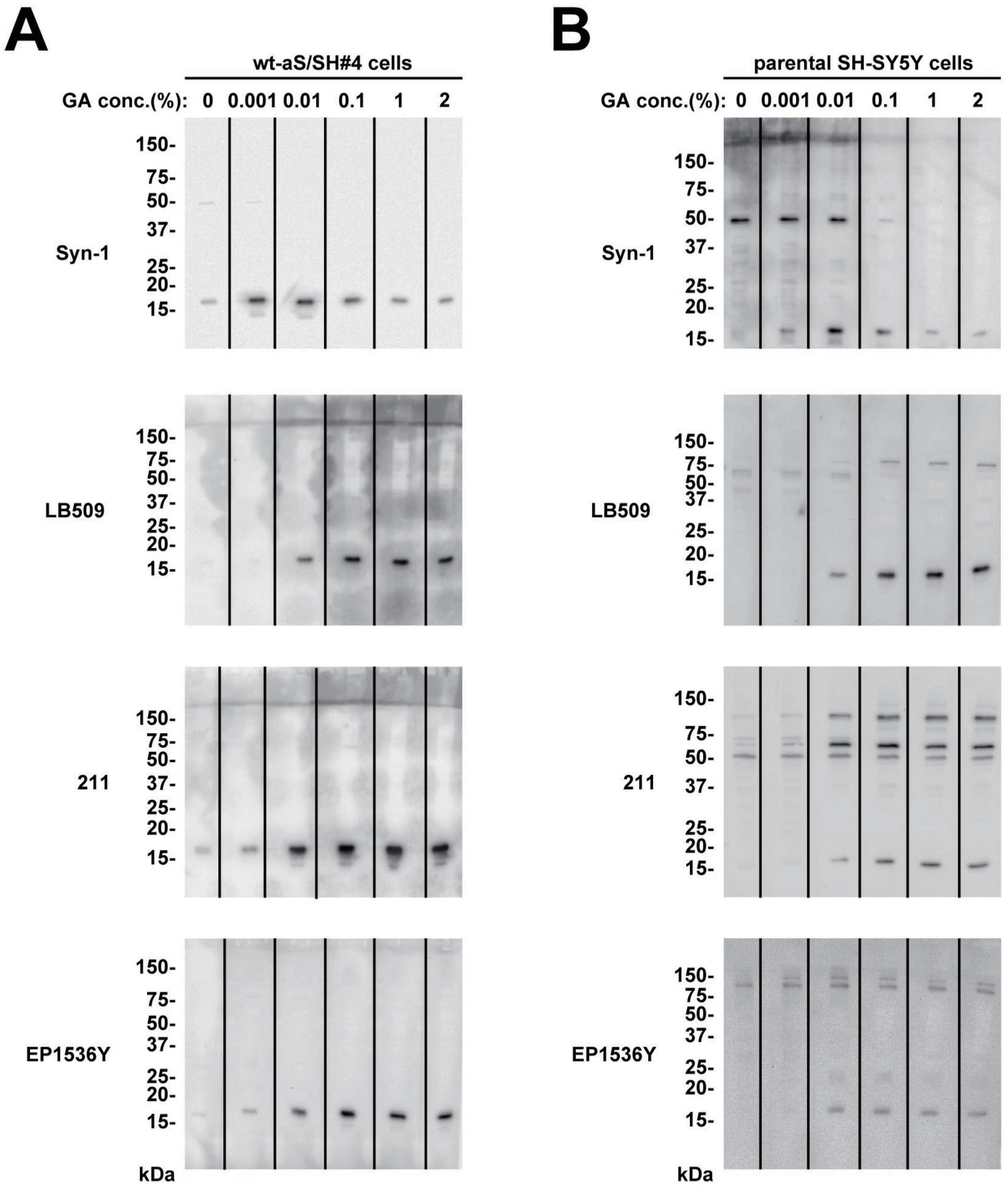

Supplementary figure S2. Full-length images of figure 2. The origin of these panels is identical with figure 2 showing western blotting with Syn-1, LB509, 211, or EP1536Y antibody.

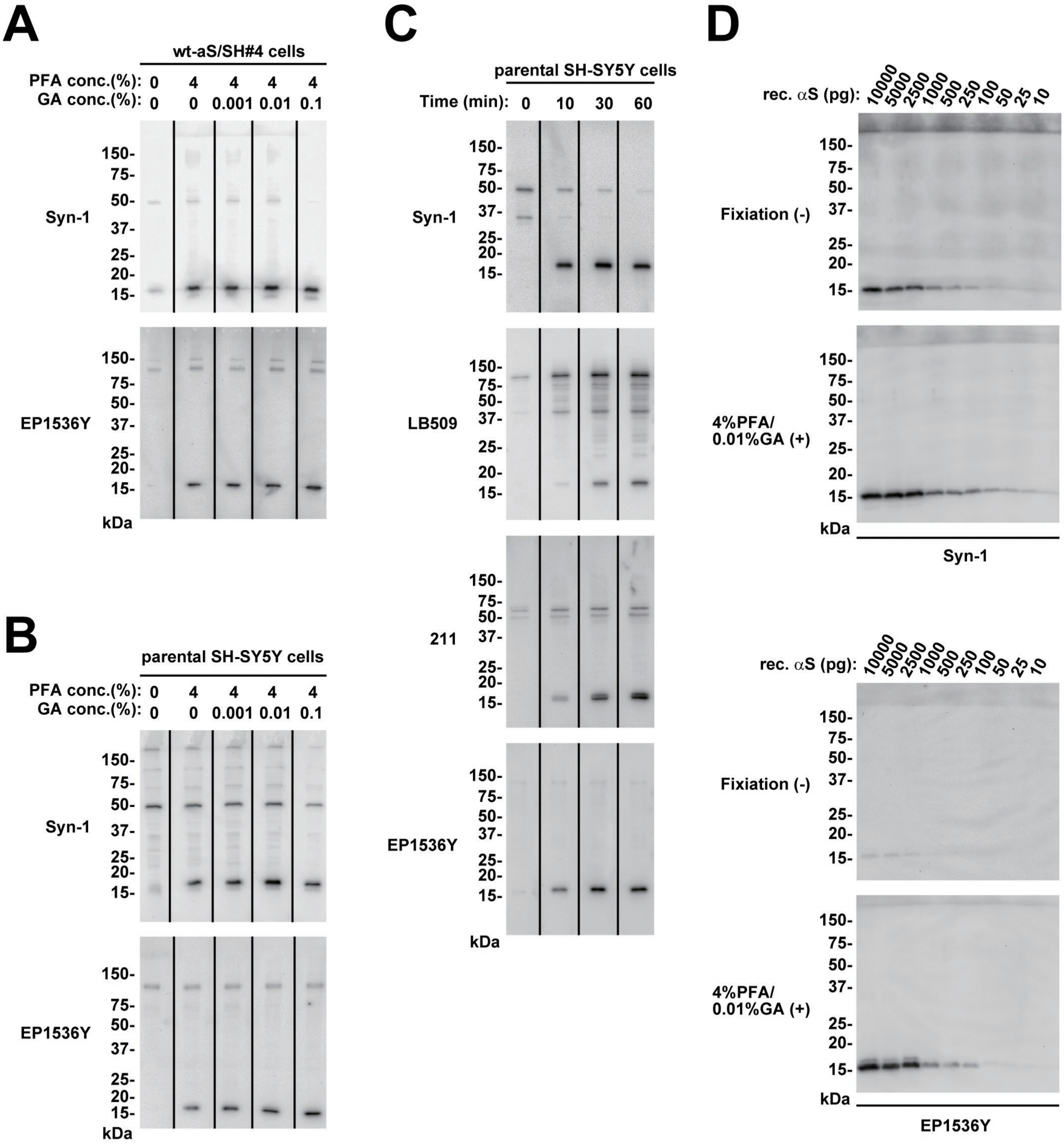

Supplementary figure S3. Full-length images of figure 3. The origin of these panels is identical with figure 3 showing western blotting with Syn-1, LB509, 211, or EP1536Y antibody.

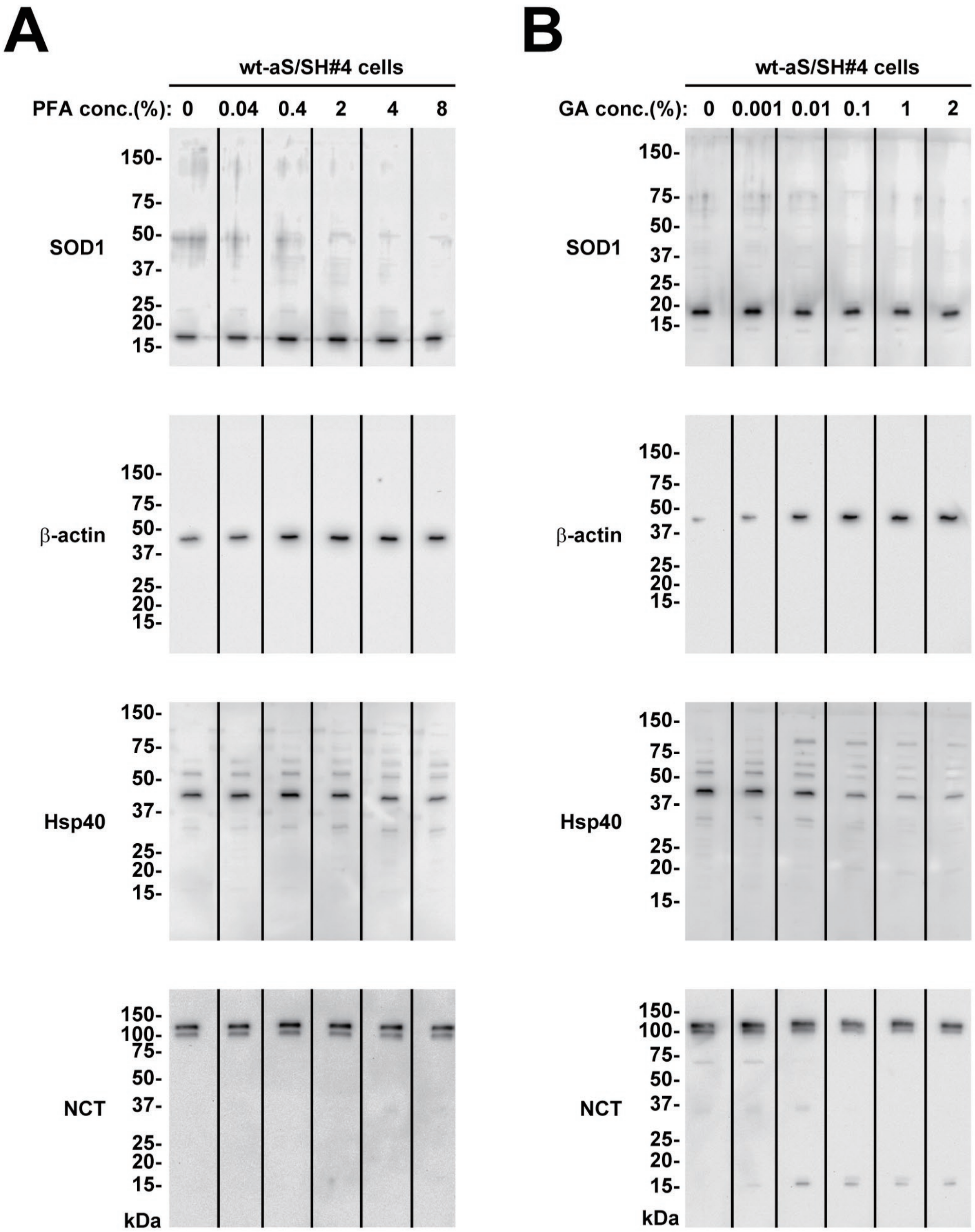

Supplementary figure S4. Full-length images of figure 4. The origin of these panels is identical with figure 4 showing western blotting with SOD1, β-actin, Hsp40, or NCT antibody.

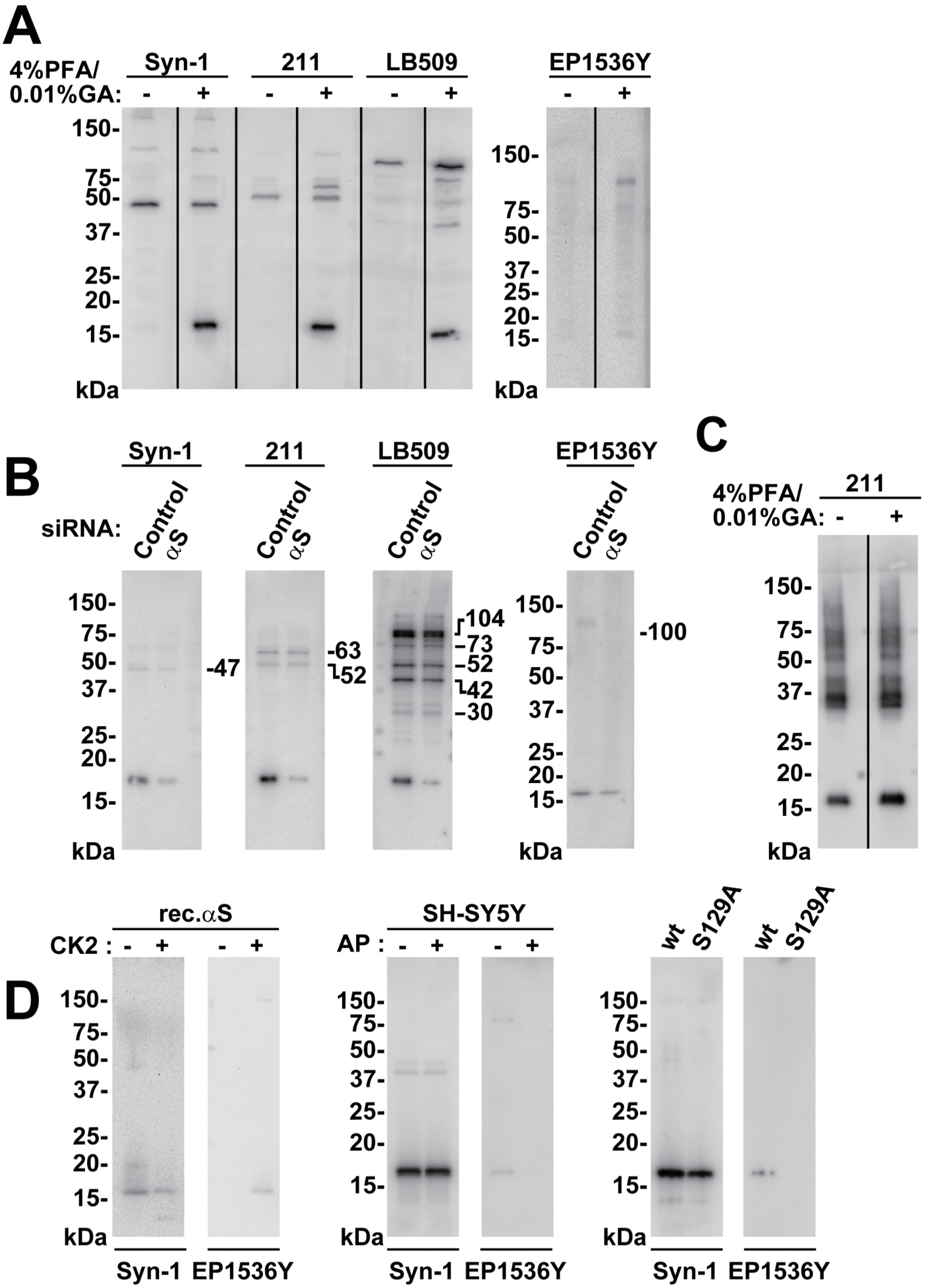

Supplementary figure S5. Different images of figure 5. We repeated independent experiments three times. These panels show the data obtained from a different experiment. The composition of panels is identical with figure 5.

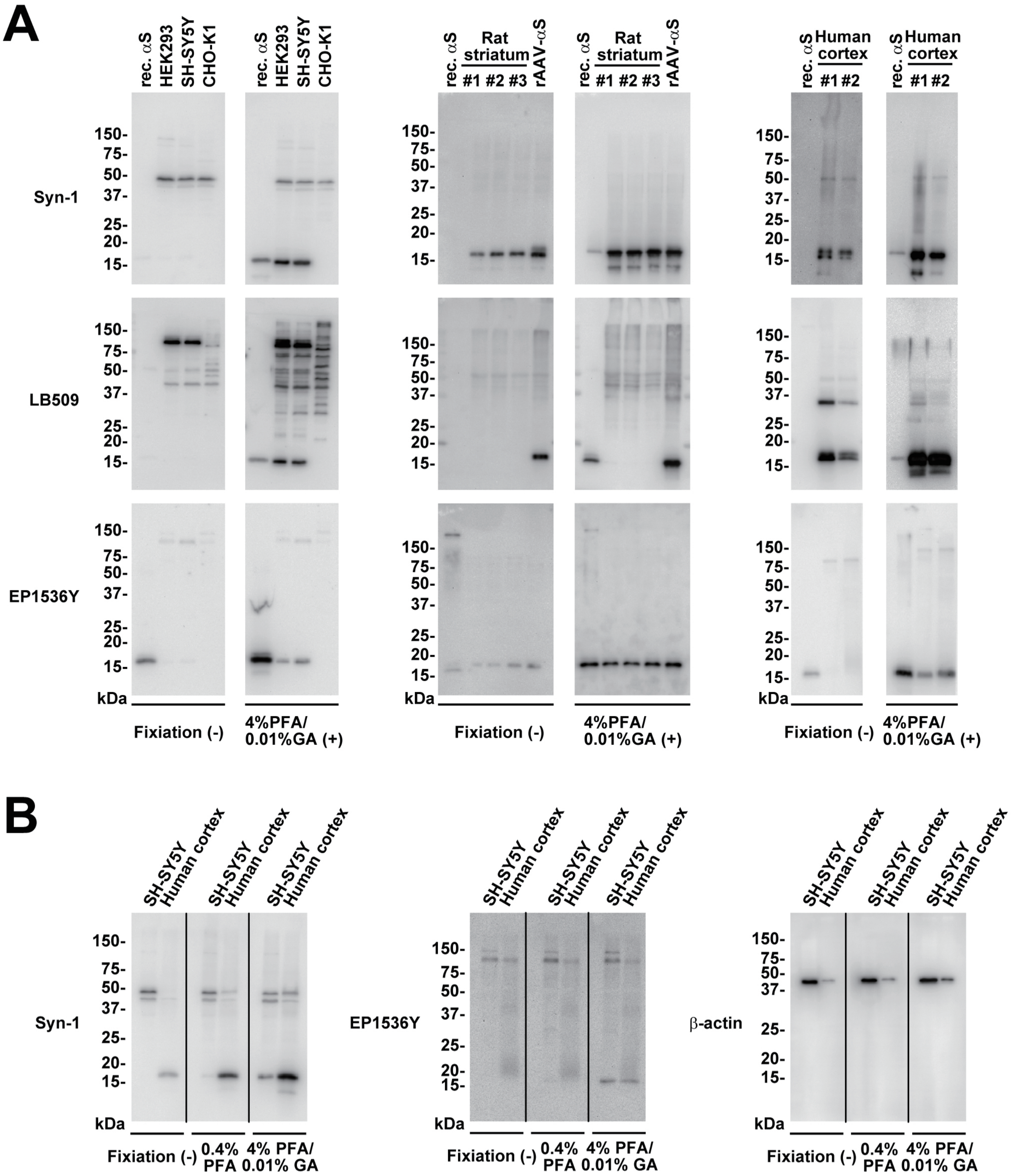

Supplementary figure S6. Full-length images of figure 6. The origin of these panels is identical with figure 6 showing western blotting with Syn-1, LB509, or β-actin antibody.

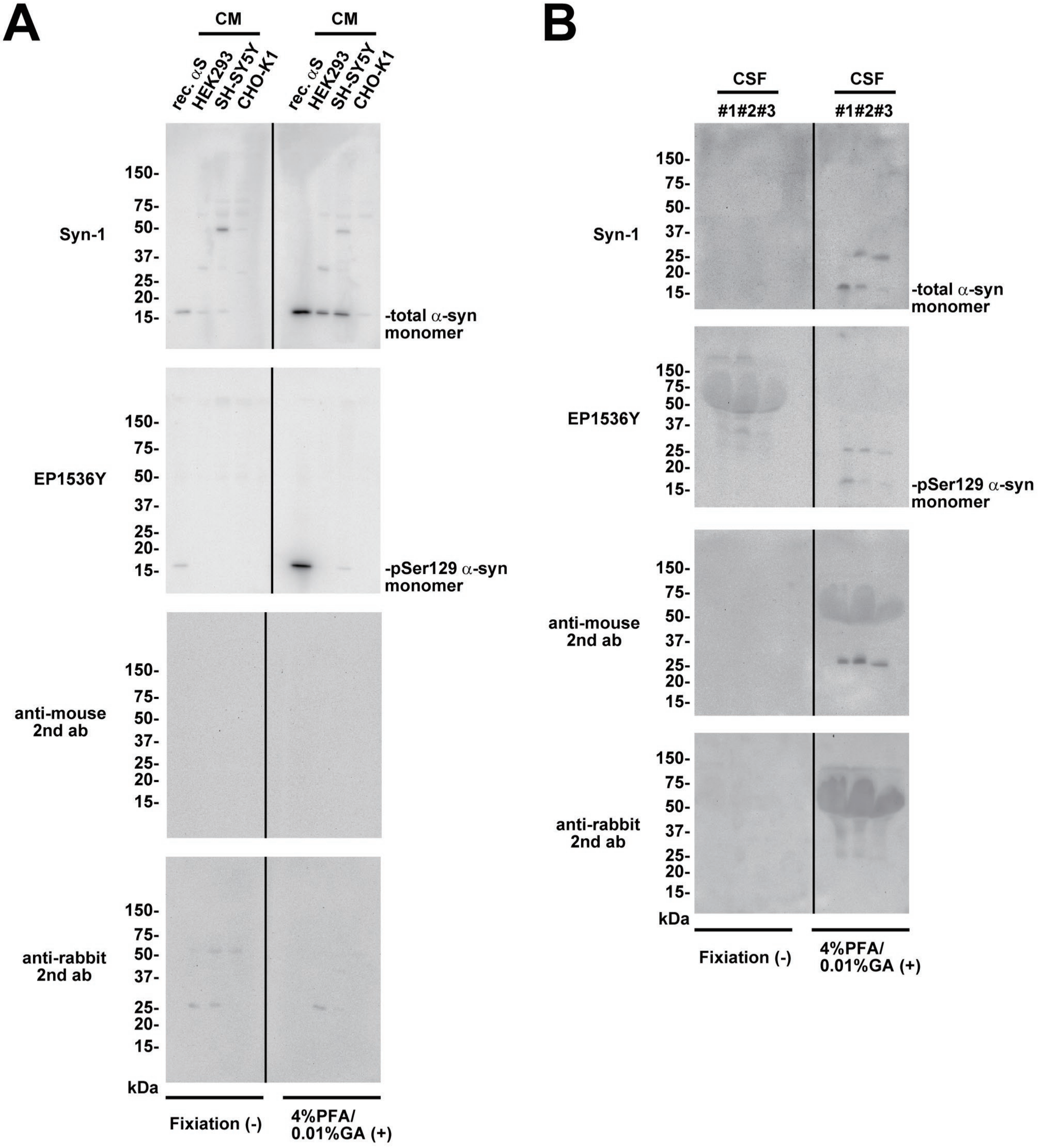

Supplementary figure S7. Full-length images of figure 7. The origin of these panels is identical with figure 7 showing western blotting with Syn-1 or EP1536Y antibody.
